# A Multiple Targeted Research Protocol for a Quasi-Experimental Trial in Primary School Children Based on an Active Break Intervention: The Imola Active Breaks (I-MOVE) Study

**DOI:** 10.3390/ijerph17176123

**Published:** 2020-08-23

**Authors:** Alice Masini, Marcello Lanari, Sofia Marini, Alessia Tessari, Stefania Toselli, Rita Stagni, Maria Cristina Bisi, Laura Bragonzoni, Davide Gori, Alessandra Sansavini, Andrea Ceciliani, Laura Dallolio

**Affiliations:** 1 Department of Biomedical and Neuromotor Science, University of Bologna, 40126 Bologna, Italy; alice.masini7@unibo.it (A.M.); stefania.toselli@unibo.it (S.T.)laura.dallolio@unibo.it (L.D.); 2 Department of Medical and Surgical Sciences, University of Bologna, 40138 Bologna, Italy; marcello.lanari@unibo.it; 3 Department of Life Quality Studies, University of Bologna, Campus of Rimini, 47921 Rimini, Italy; laura.bragonzoni4@unibo.it (L.B.); andrea.ceciliani@unibo.it (A.C.); 4 Department of Psychology, University of Bologna, 40126 Bologna, Italy; alessia.tessari@unibo.it (A.T.); alessandra.sansavini@unibo.it (A.S.); 5 Department of Electrical, Electronic, and Information Engineering “Guglielmo Marconi” University of Bologna, 40136 Bologna, Italy; rita.stagni@unibo.it (R.S.); mariacristina.bisi@unibo.it (M.C.B.)

**Keywords:** school based intervention, moderate to vigorous physical activity, public health, sedentary behavior, quality of life, physical fitness, time-on-task, cognitive function, fine and gross motor control

## Abstract

Background: Children and adolescents should perform, according to the World Health Organization guidelines, at least 60 min of moderate-to-vigorous physical activity per-day in order to avoid the risk of metabolic and cardiovascular diseases. The school represents a fundamental setting to conduct interventions to promote physical activity (PA) and contrast sedentary behaviors. Active breaks (ABs), bouts of 10 min of PA conducted inside the classroom, seem to be a good strategy to promote PA and improve classroom behavior. The aim of this study protocol is to describe the design and the assessment of the Imola Active Breaks I-MOVE study. Methods: The I-MOVE study is a school-based intervention trial, with a quasi-experimental design, performed in a primary school. It involves one experimental-group performing the intervention, focused on ABs, and one control-group. Nine main outcomes are evaluated: PA and sedentary behaviors; health related fitness; motor control development; dietary patterns; anthropometric evaluation; sociodemographic determinants; cognitive function; time-on-task behavior and quality of life. Conclusions: Results from the I-MOVE study will help to clarify the effects of incorporating ABs in the Italian school curriculum as a new public health strategy and an innovative school model oriented to the well-being of children and teachers for the best quality of school life.

## 1. Introduction

Many studies report that regular physical activity (PA) during childhood is correlated with physical, mental, emotional and social health benefits [1,2]. According to the World Health Organization children and adolescents, aged 5–17, are recommended to perform at least 60 min of moderate-to-vigorous PA (MVPA) per day in order to avoid the risk of metabolic and cardiovascular diseases [3]. Furthermore, a poor amount of PA in children and adolescents are considered as a crucial risk factor for chronic diseases in adult life [4]. However, despite these recommendations, more than half of children and adolescents worldwide do not meet the recommendation of 60 min of MVPA per day [5,6] and it is estimated that in Italy only 9.5% of boys and 2.6% of girls achieve the daily amount of PA [7]. Currently, children and adolescents spend most of their time at school [8] this environment gives access to children regardless of age, ethnicity, gender, and socioeconomic class without any distinctions, and providing an inclusive place for everyone. Therefore, the school can be considered as an effective socializing context for health promotion interventions [9,10], especially to promote children’s good lifestyles and PA [11].

For this reason, schools should provide space, time, facilities and new strategies to encourage children to be physically active, helping to reach the 60-min per day of moderate-to-vigorous physical activity guideline [12]. Unfortunately, many school settings are not providing children with adequate opportunities to become physically active and may not generate enough PA in children and adolescents. Studies report that children and adolescents in European countries spend 65–70% of school hours in sedentary behaviors [13,14].

In this frame, we propose a quasi-experimental study to assess the feasibility and efficacy of a school-based intervention based on active breaks (ABs) to promote PA within the classroom context. ABs are 10–15 min bouts of MVPA activity incorporated in the school time, performed inside the classroom context and led by teachers during the academic lessons. Incorporating active breaks in the school day has been highlighted as a positive strategy for helping children in accumulating the required amount of PA [15]. ABs are also emerging as a promising way of increasing the PA levels, and reaching positive learning outcomes [16]. As suggested by a recent systematic review with a meta-analysis, ABs interventions have positive effects in increasing PA levels and improving classroom time-on-task behavior [17]. In particular, the evidence suggests that with 10 min thrice a day for 12 weeks of an AB intervention [16] or 10–15 min once a day for 9 months [18], it is possible to get the above effects of long duration. Most studies included in this review were performed in Australia and the USA; only one study was performed in Italy [19]. The purpose of this study protocol is to outline the design, procedures and methods used in the Imola Active Breaks study (I-MOVE study). Our study is one of the first that concerns ABs intervention carried out in an Italian setting with a two-year follow-up and with a multidisciplinary team that manages the different time assessments for multiple outcomes. Furthermore, to our knowledge, the I-MOVE protocol is the first that includes HIIT exercises within the exercise protocol. The I-MOVE study could provide key recommendations for using these interventions in the organization of Italian schools setting.

### Aims of the I-MOVE Study

1. To evaluate the effects of an active breaks intervention in increasing total day and school day PA and reducing time spent in sedentary habits in children attending primary school; the relationship between objective and reported measures of PA and health-related fitness will be examined.

2. To assess the possible effect of active breaks in improving quality of life in children, stratified by anthropometric evaluation, diet habit, socioeconomic status and parent-perceived children’s quality of life.

3. To investigate the effects of active breaks as a strategy to change the time-on-task behavior and the cognitive function of children (executive functions and multisensory perception).

4. To investigate the possible effects of active breaks in improving the development of fine and gross motor control, quantified using wearable sensors.

## 2. Materials and Methods

### 2.1. Study Design

The I-MOVE study is a school-based intervention study with a quasi-experimental design (controlled studies in which exposure was assigned by the investigator without using randomization) [20]. The current project was based on a feasibility pilot study [21] and a meta-analysis on the evaluation of school-based interventions [17]. Findings from both were used to design, structure, manage and organize the I-MOVE study. This intervention trial follows the guidelines of the SPIRITS (Standard Protocol items for intervention trials) Checklist.

The I-MOVE study was endorsed by the University of Bologna (Italy).

The administration of the study was designed to allow effective collaboration and communication among different departments of the University of Bologna.

The study was approved by the University of Bologna Bioethics Committee, on the 18th March 2019 (Prot. n. 0054382 of 18/03/2019-[UOR: SI017107-Classif. III/13]). The study was conducted following the Declaration of Helsinki and approved by the school board.

### 2.2. School and Participant Recruitment

The study setting was a primary school in the city of Imola (70,075 inhabitants, Bologna, Northern Italy).

Invitation letters accompanied by an expression of interest form were sent to the principals of 4 schools of the city of Imola. One school expressed interest in participating in the project. After that, a member of our team visited the interested school to give a global view of the project in front of the relevant staff. Furthermore, a specific meeting with the headmaster was held to obtain the school’s agreement. Finally, we organized a presentation day to explain the project to the teachers. Ten teachers of 5 classes out of 15 agreed to be involved in the project. They attended a training course of 8 h to learn the basis of the project and the practical part of the active breaks intervention. The parents of the children received a short brochure describing the study, and an invitation to attend an informative meeting at school. The purpose of the meeting was to explain the intervention that would be carried out in the classrooms. At the end of the meeting informed consent was obtained from parents/tutors.

The I-MOVE study established the following inclusion criteria: (i) studying in 1st to 5th grade (aged 6–11 years), (ii) not having health problems or physical disability, which might limit the performance of ABs and (iii) having obtained informed consent of parents and permission for personal data processing.

**Figure 1:**
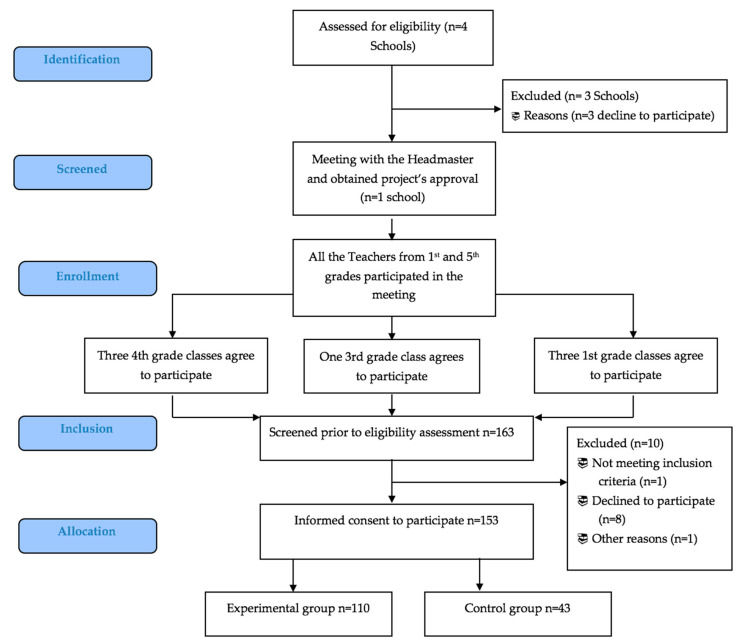
Flow chart of the I-MOVE study.

Finally, 153 children were enrolled in the study (Figure 1). The mean number of children in the classes was 22. Participants were free to retire from the study at any point without giving explanations.

### 2.3. Sample Size

Sample size was estimated considering the actigraph accelerometers as a primary outcome measure of the study. Sample size calculations were based on the pilot and feasibility study [21], which detected a mean difference of 98.1 min in weekly total MVPA, measured using the accelerometer, between the intervention group and control group.

Considering an alpha error of 0.05 and a power of at least 0.8, the minimum size of the sample was estimated as 48 participants per group, for a total of 96 participants, without considering the nested data structure. Power analysis was carried out with Clinicalc.com.

### 2.4. Intervention

The I-MOVE protocol was developed and tested, based on a previous pilot and feasibility study [18]. Some exercises of the I-MOVE protocol were implemented and suggested by the teachers of the previous Valsamoggia study [21].

The experimental groups performed the I-MOVE protocol thrice a day without a fixed time: usually two breaks in the morning and one in the afternoon every weekday. Active breaks were performed in addition to the normal recess breaks time. In particular, children got up from their chair, placed it under the desk to ensure their safety and positioned themselves to the side or behind their seat, depending on the type of exercises. Approximately every child needed 1-m diameter of free space around to perform exercises in safety.

Each active break consisted of 10 min, divided into three different parts (Table 1). The first part, called “warm-up” (2 min), focused on cardiorespiratory and mobility exercises to prepare children to increase their motor activity intensity. In the central part, called “tone up” (5 min), teachers conducted exercises with high-intensity interval training (HIIT), consisting of 40 s of vigorous PA alternated with 20 s of recovery, with a specific focus on coordination and balance. During the tone-up the children could experiment with and learn basic motor skills such as jumping and throwing. In the last part of the active break, called “cool-down” (3 min), children performed stretching, relaxation and breath control exercises. Following these exercises the children were recharged to restart the academic lesson.

The teachers could decide together with the pupils which daily exercises they liked to perform, trying to use the whole range of exercises and varying them as much as possible to keep up the enjoyment and motivation of the children. Furthermore, this modality allowed teachers to adapt the active breaks protocol according to the needs of their class, for example not using contact exercises in particular cases. Clearly, the I-MOVE protocol, performed for 10 min three times a day for every day of the week, takes time away from the curricular lessons. However, these interventions appear to benefit the classroom behavior by improving children’s attention and making teachers easier to do their jobs [22,23].

Active breaks are also reported by the teachers themselves as a useful strategy for optimizing the time dedicated to academic lessons in particular because active breaks are able to energize children who have difficulty in maintaining concentration during daily school activities [19,21].

### 2.5. Data Collection and Outcome Measures

During the Imola Active Breaks Study the following outcomes will be evaluated: (1) PA and sedentary behaviors; (2) health related fitness; (3) motor control development during fine and gross (locomotor) tasks; (4) dietary patterns; (5) anthropometric evaluation; (6) sociodemographic and early determinants; (7) cognitive Function; (8) time-on-task behavior and (9) quality of Life.

As shown in Table 2, all the measurements will be evaluated at baseline T0 (October 2019) and will then be assessed at mid-intervention T1 (October 2020) and at the end of the intervention T2 (June 2021) to investigate group differences and changes over time. The process evaluation will performed 6 months after the end of the intervention T3 (December 2021.)

The comparisons between T0, T1 and T2 will allow the research to determine the timing of any long-term effect of ABs and whether the effects of ABs are cumulative in time or reach a ceiling after T1 (for example, if there are cumulative effects, then performance should reveal a pattern T0 < T1 < T2; by contrast, if they reach positive effects in a few months and then stabilize, then performances at T1 and T2 should not differ).

Moreover, we planned an assessment at the mid-intervention to measure an acute effect before the daily active breaks and immediately after the 10-min bout for cognitive and time on task outcomes. During this specific mid-intervention we will monitor the amount of PA performing an active break, 10 min thrice a day, during a school day for a week. This assessment will be focused on cognitive function and time-on-task behaviors, since these could be more affected after acute exercises than chronic ones, as shown for some cognitive functions in the literature [24,25,26].

#### 2.5.1. PA and Sedentary Behavior

The time spent on physical activity and sedentary behavior will be calculated through actigraph accelerometers (ActiLife6 wGT3X-BT). The actigraph accelerometer models GT3X (ActiGraph LCC: Pensacole, FL, USA) is used to monitor objectively the daily PA and sedentary behavior over seven consecutive days. We examined the accelerometer data through ActiLife 6.13.3 software (ActiGraph LCC: Pensacole, FL, USA).

The epoch length will analyze to 10 s to allow a more detailed estimate of PA intensity [27]. The screening and data processing procedures to evaluate time spent in sedentary, total PA and PA at different intensities are consistent with previous studies on children and adolescents [28,29,30]. The children will wear the accelerometers over a seven-day period (five weekdays and two weekend days), only to be removed when bathing, swimming and showering. Accelerometers are attached around the waist with an elastic belt, in line with the existing literature [31].

We will analyze the accelerometer’s data only when children complying with some specific inclusion criteria: having worn the accelerometer on at least 3 weekdays and 1 weekend day, and for at least 10 h every day (including sleeping hours). Minutes spent in physical activity (light, moderate and vigorous) per day will be calculated using the Evenson cut points [32].

PA will also be evaluated with the physical activity questionnaire for children (PAQ-/C).

The PAQ-C is a self-administered, 7-day recall questionnaire, with 9 items scored on a five-point scale. This instrument provides a final composite activity score, by taking the mean of the 9 items. This questionnaire has been shown to be valid and reliable [33].

#### 2.5.2. Health-Related Fitness

Health-related fitness will be assessed by the following tests: 6-min running test, 6-min walking test (used only in 5–6 year-old children, first grade), Harre test, standing long jump test and shuttle run 4 × 10.

The 6-min running test (6MRT), derived from Cooper’s 12-min test, consists of performing the maximum running distance possible, also walking when tired, for a time of 6 min. At the end of the test, the meters covered in the given time are considered. The test is validated for preschool [34] and school-aged children [35]. The 6-min walking test (6MWTs) was conducted using ATS guidelines (2002) [36]. All children will receive the same instructions before undertaking the walk test. The children are asked to walk up and down at their best pace but not to run or race. The children will be explained that it was not a competition. During the performance, no indications such as “Slow down” or “Go faster”, were given, except for encouragement (e.g., “You are doing great” and “Keep going”) [37,38]. Only 5–6 year-old children from the whole sample performed this test, as it is easier and more adaptable to this age group. Both the 6 MRT and the 6 MWT are a reliable test to assess the cardiorespiratory fitness in children.

Standing long jump (SLJ) or standing broad jump is a good test to assess lower body strength, and it could be considered as a general index of upper and lower body muscular fitness in youth [39,40]. The participant stands behind the starting line, with feet together, and pushes off vigorously and jumps forward as far as possible. The distance is measured from the take-off line to the point where the back of the heel nearest to the take-off line lands on the mat or non-slippery floor. The test will be repeated twice, and the best score retained (in cm).

The Harre circuit (HC), a dexterity’s test, is a timed instrument based on simple and dynamic movements repeated in three spatial directions: right, forward and left [41,42,43].

The shuttle run 4 × 10 test (4 × 10 SRT) [44] measures agility, speed and coordination while running between two lines drawn on the floor 10m apart to pick up small blocks. The children ran as fast as possible from the starting line to the other line and return to the starting line, crossing each line with both feet every time. This test will be performed twice, covering a distance of 40 m (4 m × 10 m). Each time the children crosses any of the lines, he/she had to pick up (the first time) or exchange (second and third time) a sponge that is previously placed behind the lines. The stopwatch is interrupted when the children cross the end line with one foot.

#### 2.5.3. Motor Control Development During Fine and Gross (Locomotor) Tasks

Gross- and fine-motor competences will be assessed using an instrumented approach based on wearable inertial sensors [45,46]. The selected instrumented approach allows the quantitative analysis of motor competence characteristics (e.g., automaticity, complexity and regularity) and their longitudinal monitoring as related to age and/or motor development.

For gross-motor competence 3 inertial sensors (OPAL; APDM: Portland, OR, USA) will be attached, using elastic Velcro bands, to the lower shanks (above lateral malleolus) and lower back (at L5 level) of each child over clothes, and 3D acceleration and angular velocity is acquired from each sensor during natural and tandem gait along a 15 m straight path. Each child walks 3 times back and forth normally (for a total of 45 m) and 1 time in tandem (along the straight line, positioning the heel of the front foot in contact with the toes of the back one), prior to data collection, all participants are allowed to perform a tentative trial (10 TW strides) to ensure they understand the tandem gait instructions.

For the assessment of fine-motor competence, the 3 inertial sensors (OPAL; APDM: Portland, OR, USA) will be attached to the wrists (dorsal aspect) and lower back (at L5 level), and 3D accelerations and angular velocity will be acquired during the Placing bricks test [47]. Each child, sitting at a desk, attached 18 1 × 2 Duplo bricks one at a time onto a 6 × 12 Duplo plate using one hand, then the other for the second trial; blocks will be positioned on the side of the plate corresponding to the hand to be used, while the opposite hand was kept on the table or used to stabilize the plate according to child’s preference. For all trials data are acquired at 128 Hz sampling frequency.

Temporal parameters, variability, regularity, motor complexity, stability and rhythmicity calculated from acquired motion and represented in a graphical polar plot [46] allow one to qualitatively characterize different areas of motor control performance.

#### 2.5.4. Dietary Patterns

The ZOOM-8 study [48] is conceived to investigate more in depth the dietary habits and physical activity of Italian primary school children, and the role of the health services in geographic areas with different levels of childhood overweight and obesity, as shown in the Italian surveillance system named “OKkio alla SALUTE”.

In the ZOOM-8 study, the anthropometric measures of children are taken and their parents fill in two questionnaires, one general questionnaire including general information along with lifestyle questions (i.e., amount of physical activity, time spent at the screen, sleeping hours, family composition, etc.) and a semiquantitative food frequency questionnaire (FFQ), built up following the methodology described and validated by Willett [49], consisting of 53 commonly used food items categorized into 11 food groups [50].

We will use the same two questionnaires, asking the children’s parent participating in the study to complete them and providing specific instructions on filling them in at home. Frequency response categories for all food portions ranged from the number of times per day, per week, per month, per year to never. Manufactured products will be reported as number (or fraction) of units consumed.

#### 2.5.5. Anthropometric Evaluation

Six anthropometric characteristics (height, weight, waist and hip circumferences, triceps and subscapular skinfold thicknesses) will be collected according to standardized procedures [51,52]. In particular, height is measured to the nearest 0.1 cm using a portable stadiometer (SECA 217, SECA: Hamburg, Germany). Body weight is measured to the nearest 0.1 kg (light indoor clothing, without shoes) using a calibrated electronic scale (SECA 877: Hamburg, Germany). Waist circumference (WC) and hip circumference (HC) are measured to the nearest 0.1 cm with a non-stretchable tape (GPM measuring tape; DKSH Switzerland Ltd.: Zurich, Switzerland): WC is measured between the lowest rib and the iliac crest and HC at the widest part of the hip. Triceps (TSF) and subscapular (SSF) skinfold thickness is measured to the nearest 0.1 cm on the left side with a Lange caliper (Beta Technology Inc.: Santa Cruz, CA, USA). TSF is measured midway between the tip of the acromion and olecranon processes, while SSF raising an oblique skinfold below the inferior angle of the scapula at 45° to the horizontal plane following the natural cleavage lines of the skin. TSF and SSF were evaluated according to the Frinsancho cutoff (2008) [53].

BMI will be calculated as weight (in kilograms) divided by the square of height (in meters). This index will be used to assess the weight status of each participant according to Cole cutoff values by sex and age [54,55]. Consequently, we will calculate the BMI z-score.

Waist/height ratio (WtHR) will be calculated and children will be stratified into two categories (≤0.5 and >0.5); the value of 0.5 is chosen as the cut-off of cardiovascular risk [56,57,58].

Body composition parameters (percentage fat (%F), fat mass (FM) and fat free mass (FFM)) will be calculated using the skinfolds equations of Slaughter et al. (1988) [59] and the cutoff for %F by Laurson (2011) [60] is used to identify subjects with lower or higher than recommended values.

#### 2.5.6. Sociodemographic Determinants.

Parents or guardians will fill out a questionnaire (inside the ZOOM8 Questionnaire) [49,50] about sociodemographic factors (e.g., sex, age, birth date, place of birth and type of work) as well as their habitual PA and sedentary habits.

#### 2.5.7. Cognitive Function

Two cognitive tasks will be administered: one task investigates the executive functions (i.e., working memory), which are sensitive to exercise, the other investigates multisensory perception.

Verbal working memory will be assessed by means of the backward digit span, a subtest of the Wechsler intelligence scale for children (WISC-IV) [61]. The task involves the verbal presentation of digit series and requires children to repeat the series in reverse order. The score will be calculated as the highest number of correct digits remembered.

Multisensory perception will be investigated using the sound-induced flash illusion, SIFI [62,63]. The SIFI consists of the illusory perception of two flashes when one flash is presented simultaneously with two (beep) sounds. Susceptibility to the SIFI is considered an indicator of efficient multisensory processing [64,65,66]. In this task, 1 or 2 flashes (the number of which has to be reported) is presented together with 1 or 2 task-irrelevant sounds. Different multisensory congruent (1flash/1beep, 2flashes/2beeps) or incongruent (1flash/2beeps) conditions are presented. The stimulus onset asynchrony (SOA) varies amongst trials (with SOAs of 70, 110, 150 and 230 ms) in the 1flash/2beeps and 2flashes/2beeps conditions.

#### 2.5.8. Time-On-Task Behavior

We will structure a self-administered active breaks questionnaire for teachers to monitor a possible change in the behavior of the children. The teacher’s questionnaire will include different domains regarding the satisfaction, the feasibility, the effectiveness and the organizing of the ABs. The questionnaire will include items investigating potential changes in the time-on-task behavior, attention and wellbeing of the children, and their personal attitude in handling, implementing and performing ABs. We also perform a children’s self-administered active breaks questionnaire to explore satisfaction, feelings and pleasure in performing ABs.

#### 2.5.9. Quality of Life (HRQoL)

The Pediatric Quality of Life Questionnaire 4.0 (PEDsQL) [67] will be used to measure the health-related quality of life in the children (HRQoL) and to assess important determinants of health such as daily activities, physical health, social interactions and emotional well-being. We will obtain the self-reported children’s HRQoL total score and the parents’ perceived children’s HRQoL total score.

#### 2.5.10. Process Evaluation of Children and Teachers

At the 6-month follow-up (T3), children and teachers will be invited to take part in focus groups meetings. These focus group sessions will allow both children and teachers to give their feedback about the active breaks intervention. Firstly, we will conduct focus groups with all the teachers of the experimental groups that performed the I-MOVE protocol. This session will be fundamental to have feedback on the efficacy, feasibility and adherence to the protocol by teachers. Secondly, we will involve teachers of the control group to be part of a focus group to understand their attitude for a future involvement in the experimental part of the project and the reason why they chose not to perform active breaks. This second focus group will be essential to outline barriers and facilitators for participation in these health promotion interventions (Appendix A). It seems very important to understand, together with the teachers, the feasibility of the project and its positive integration into daily school activities, without negative effects on children’s learning in other subjects (Math, Science, Italian and so on). In other words: the project is easily achievable and does not create problems or slowdowns in teaching other disciplines, rather it reveals positive effects on the participation and involvement of children in daily school life. This is the real wall to break down, which often prevents teachers from experimenting with new school day models.

After these two focus groups, children of the experimental classes will be involved in a discussion session. We will organize a meeting for each class that performed the I-MOVE protocol to obtain the children’s feedback about the intervention. This meeting will be managed and conducted by a researcher of our team qualified for qualitative research methods (expert in developmental psychology and education) with the supervision of class teachers (Appendix B).

Obviously, the educational network that surrounds the child also involves parents in what is called the educational triangle. Parents will also be invited to collaborate through questionnaires and discussion during the annual meetings with teachers. In all the scheduled focus groups a moderator will manage the meeting entirely, a supporter figure will help the moderator in conducting the discussion and an observer will ensure that the meeting is carried out in the best possible way.

Qualitative research together with quantitative research represents a complete strategy to measure and understand phenomena related to health [68,69].

### 2.6. Data Analysis

Data will be recorded electronically on a secure file storage system and safeguarded by a password. Data will be anonymized by assigning a unique identification number to each pupil.

#### 2.6.1. Quantitative Analysis

The statistical analysis will be performed using SPSS (Statistical Package for Social Science) (SPSS Inc. Chicago, IL, USA) The data will be reported as the mean and standard deviation for both experimental and control groups and for the T0, T1 and T2. We will use the Student’s *t*-test, (for parametric variables) the Mann–Whitney test, (for non-parametric variables) and the Chi-square test (dichotomous ones) to compare the general characteristics between the groups. Differences between the two groups from baseline to follow-up will be analyzed using a one-way ANOVA and ANCOVA adjusted for covariates. We will consider the results statistically significant only if the *p* value will be lower than 0.05. We will calculate the effect size to examine the magnitude of any mean differences.

#### 2.6.2. Qualitative Analysis

The research team includes different professional figures including psychologists and pedagogists. They will manage the qualitative data collected through interviews, questionnaires and focus groups conducted by qualified and competent researchers in qualitative research. The qualitative analysis will provide support in evaluating the time on task behavior outcomes with semistructured questionnaires. We will use qualitative methods, such as thematic analysis techniques and grounded theory approach [70], to organize and manage all the focus groups in order to obtain fundamental information for the implementation of the I-MOVE protocol.

Focus groups will be audio-recorded, transcribed verbatim and anonymized before being coded. The analysis will be carried out using the “Nvivo version 12 Plus” software (version12; QRS International-Melbourne, Australia).

### 2.7. Harmonization and Standardization

An operation manual will be prepared to be followed by all participants involved in the evaluation. This manual contains all the data collection methodology and a specific description of all instruments used. All teachers involved in the active break practice will be invited to follow a manual where all the exercises are explained, for all the classes involved. Each teacher will have the opportunity to adapt the exercises to his/her class and to add elements if necessary. The protocol foresaw 3 daily breaks lasting ten minutes. Every two months, teachers will be asked to report whether they will be able to comply with the frequency and intensity established by the protocol or if changes will be made for causes external or internal to the class.

### 2.8. Dissemination of Project Findings

To disseminate the findings, we will organize dissemination events (e.g., school seminars for teachers and parents, specialized participatory seminars for all school children, university seminars and proposals for teacher training courses). These will be open to all stakeholders, we will present the main findings of the I-MOVE study, and we will provide further information on how schools could implement the program. Part of the dissemination will be carried out through articles in educational magazines and scientific journals. The results will be disseminated in peer-reviewed publications to reach the scientific community and policy makers. Moreover, we planned to publish in an accessible magazine to better disseminate the results.

## 3. Discussion

Results from the Imola Active Breaks Study will be useful to clarify and objectivize the effects of incorporating active breaks in Italian school. The Italian guidelines on PA suggest, in the school setting, the importance of supporting the tendency of children and teenagers to move [71]. Thus, results from this type of research could provide new strengths of evidence for recommendations and facilitate knowledge transfer in to the practice. Furthermore, they could support ABs as a new public health strategy for an innovative school model oriented to the well-being of children and teachers for the best quality of school life. Recent studies have stated that a poor amount of PA in children and adolescents are considered as a crucial risk factor for chronic diseases in adult life [4]. Moreover, the 2016 data from the OKkio HEALTH [72] surveillance system reported 21.3% overweight children and 9.3% obese children, including 2.1% severely obese children. Due to the fact that physical inactivity has a heavy negative impact on health system direct costs, it is becoming increasingly necessary to propose sustainable and feasible interventions to promote physical activity. Many studies were performed in the school context to evaluate the potential effects of classroom-based PA interventions [73,74,75]. Incorporating active breaks throughout the school day has been reported as a promising way for children to reach the recommended levels of PA [15]. Furthermore, active breaks conducted by trained teachers inside the classroom are rising as a good strategy for increasing the PA levels and achieving positive learning outcomes [16]. However, as reported by Wassenaar et al. [76], the literature focused on the school-based intervention needs long term follow-up assessments, exploring the effect of different PA characteristics (intensity and duration), monitoring the PA dosage that participants receive, and analyzing the influence of other determinants (e.g., sex, ethnicity and socioeconomic status).

To our knowledge, this is the first study in Italy that investigates the effectiveness of an active breaks intervention with a multidisciplinary outcomes analysis and a long-term follow-up. Our hypothesis is that the results of the I-MOVE study could provide evidence firstly, from a perspective of health promotion, since this school-based intervention could help children to reduce sedentary behavior [16] and to improve the quality of their life. Secondly, from an educational point of view, it seems that active breaks could be considered as an effective instrument to improve classroom behavior, as well as impacting on attention and cognitive function [19,21]. Finally, findings from the I-MOVE study may provide evidence from a scientific point of view to show how this type of intervention could be an effective primary preventive strategy to be implemented in the school context.

## 4. Conclusions

This study protocol describes the design, the procedures and the methods used in the Imola Active Breaks Study (I-MOVE). The future results of the I-MOVE study could demonstrate how active breaks can affect various aspects of school life from a multidisciplinary point of view and how this intervention can in all respects be included in the primary school curricula.

## Figures and Tables

**Table 1:** Components of I-MOVE protocol.

Phase	Aim	Examples of the Type of Exercises	Duration
Warm-up	Cardiorespiratory and mobility exercises	“The chair march”: The children all stand up and move their chairs sideways, waiting for the teacher’s commands. They begin to march, raising their knees well and resting their toes on the chair, without pushing upwards. Progressively, they also combine the movement of the legs with the alternating movement of the arms.	2 min
Tone-up	High-intensity interval training (HIIT) exercises	HIIT Animal jumps: Children scattered around the classroom have to jump like frogs for 40 s then rest for 20 s and repeat the exercise 2 times, then they jump like kangaroos for 40 s with 20 s break to be performed 2 times.	5 min
Cool-down	Breath and relaxation exercises	The imaginary balloon: Children must inflate an imaginary balloon by inhaling and exhaling, mimicking the progressive expansion of the balloon with the widening of their arms. We ask for a very slow and long exhalation twice as long as the inspiration.	3 min

**Table 2:** The Imola Active Breaks Study data collection and outcome measures.

Outcome Measures	Baseline October 2019 (T0)	Mid-Intervention October 2020 (T1)	End of Intervention June 2021 (T2)	6 Months After End of Intervention December 2021 (T3)
Personal information (Age, Country)	√		√	
PA and sedentary behavior (accelerometer)	√	√	√	
Total reported physical activity Questionnaire	√	√	√	
Health related fitness	√	√	√	
Motor control development	√	√	√	
Dietary patterns	√	√	√	
Anthropometric evaluation	√	√	√	
Sociodemographic and early determinants	√		√	
Cognitive Function	√	√	√	
Time-on-task behavior (Teachers and children self-administrated questionnaire)		√	√	
Quality of Life	√	√	√	
Process evaluation focus group with children				√
Process evaluation: focus group with teachers				√

## Appendix A

*General Aims of the Focus Group (with All the Teachers)*

1. Knowing their attitude concerning physical activity in general;
2. Knowing their attitude in respect of the practice of active breaks;
3. Knowing if something has changed in their attitude with respect to active breaks after having practiced them for all the intervention period.

*Factors and/or Barriers to the Practice of Active Breaks (with All the Teachers)*

1. Factors related to us (barriers, limitations and points of strength. For example: a lack of personal skills, low trust and confidence in the proposed intervention);
2. Factors related to children (such as difficulties in handling them and fear of accidents);
3. Factors related to the environment (availability of space, inadequacy of space and not very stimulating exercises).

Finally, we will try to identify together hypothetical solutions and strategies that can be implemented to make this active breaks intervention more feasible, sustainable and adaptable.

## Appendix B

*General Aims of the Focus Group (with All the Children Divided by Class)*

1. Knowing their attitude concerning physical activity in general and sport practiced;
2. Knowing their attitude in respect of the practice of active breaks;
3. Knowing if something has changed in their attitude toward physical activity after having practiced active breaks for all the intervention period.

*Factors and/or Barriers to the Practice of Active Breaks (with All the Children Divided by Class)*

1. Factors related to active breaks protocol (barriers, limitations and points of strength. For example: a lack of fun and uninvolving games);
2. Factors related to themself (such as difficulties in interacting with other children and difficulties in managing the times of active breaks);
3. Factors related to the environment (small classroom’s space and too much noise).

Finally, we will try to identify together hypothetical solutions and strategies that can be implemented to make this active breaks intervention more feasible, sustainable and adaptable to children’s needs and requirements.
